# Meta-analysis and The Cochrane Collaboration: 20 years of the Cochrane Statistical Methods Group

**DOI:** 10.1186/2046-4053-2-80

**Published:** 2013-11-26

**Authors:** Joanne E McKenzie, Georgia Salanti, Steff C Lewis, Douglas G Altman

**Affiliations:** 1 School of Public Health and Preventive Medicine, Monash University, Melbourne, Victoria, Australia; 2 Department of Hygiene and Epidemiology, University of Ioannina School of Medicine, Ioannina, Greece; 3 Edinburgh MRC Hub for Trials Methodology Research, Centre for Population Health Sciences, University of Edinburgh Medical School, Edinburgh, UK; 4 Centre for Statistics in Medicine, University of Oxford, Oxford, UK

## Abstract

The Statistical Methods Group has played a pivotal role in The Cochrane Collaboration over the past 20 years. The Statistical Methods Group has determined the direction of statistical methods used within Cochrane reviews, developed guidance for these methods, provided training, and continued to discuss and consider new and controversial issues in meta-analysis. The contribution of Statistical Methods Group members to the meta-analysis literature has been extensive and has helped to shape the wider meta-analysis landscape.

In this paper, marking the 20^th^ anniversary of The Cochrane Collaboration, we reflect on the history of the Statistical Methods Group, beginning in 1993 with the identification of aspects of statistical synthesis for which consensus was lacking about the best approach. We highlight some landmark methodological developments that Statistical Methods Group members have contributed to in the field of meta-analysis. We discuss how the Group implements and disseminates statistical methods within The Cochrane Collaboration. Finally, we consider the importance of robust statistical methodology for Cochrane systematic reviews, note research gaps, and reflect on the challenges that the Statistical Methods Group faces in its future direction.

## Background

Although there are earlier examples of statistical combination of data from multiple studies, the practice began in earnest in the 1970s in the social sciences, and the term meta-analysis was coined by Glass as recently as 1976 [1]. Systematic reviews, including statistical synthesis of the results of multiple studies, are now commonplace in medical research, notably through the work of The Cochrane Collaboration. Yet it is only about 30 years since these studies began to appear in medical journals.

Methodological rigour was a key principle of The Cochrane Collaboration from its inception two decades ago. Accordingly, statistical methods have always been seen as critically important. In this article, we provide a historical account of the beginnings of the Statistical Methods Group (SMG) of The Cochrane Collaboration and its subsequent work, and reflect on the challenges that the group faces in the future. Many individuals and organisations outside the Collaboration have made significant contributions to the development of statistical aspects of systematic review methods in health care; however, in this paper, we restrict our focus to contributions of the SMG and its members.

## History of the Statistical Methods Group

A key publication pre-dating the founding of The Cochrane Collaboration was the two volume *Effective Care in Pregnancy and Childbirth*, which included a long chapter on methods for synthesising evidence from multiple studies (now called systematic reviews), including methods of meta-analysis [2]. It was recognised, however, that the field was in its infancy and there were many unresolved issues. Accordingly, the first Cochrane meeting on statistics was held at the UK Cochrane Centre in Oxford in July 1993, masterminded by Iain Chalmers and co-chaired by Ken Schulz and Doug Altman. This meeting took place just one week after a one-day meeting in London on ‘Systematic Reviews’, organised jointly by the *British Medical Journal* and the UK Cochrane Centre, which led to a book of the same name [3].

Although the statistics meeting predated (by a few months) the creation of The Cochrane Collaboration, the goal of reviewing the literature on healthcare interventions across specialties was already clear. There were 16 participants, most of whom were statisticians. The workshop was convened to develop guidelines on statistical methods for data synthesis. The hope was that the meeting would identify areas where there was reasonable consensus, and those which caused debate and could be the subject of further research. Opening the meeting, Andy Oxman described the need to develop guidelines for people preparing systematic reviews. His suggestion that it was desirable for the statistical methods to be consistent across reviews as far as possible was endorsed by most participants. It was agreed that the planned Cochrane Collaboration software should have explicit default methods of analysis and presentation but that reviewers be able to override these. Areas where there was much discussion but a lack of consensus included the use of odds ratio or relative risk (risk ratio) for meta-analysis of binary outcomes and the use of fixed or random effects analyses in the presence of heterogeneity. The minutes and list participants from this meeting are available in Additional file 1 - Oxford 1993 Workshop Report.

Following on from the meeting, Doug Altman and Ken Schulz became the founding co-convenors of the Statistical Methods Working Group (SMWG), as it was initially called. (The term ‘working’ was later dropped from the names of all Cochrane Methods Groups.) The SMWG produced an initial list of ‘possible research topics’ , shown in Table 1. They noted that “This list indicates some areas where either there is no consensus as yet about the best approach or where inadequate information is available. It should not be taken as an inclusive list of all intended topics of investigation.” From the 1993 recommendations onwards, the SMG has had a major influence on the specification of The Cochrane Collaboration software, especially in relation to meta-analysis, as discussed below.

**Table 1:** Research topics proposed in 1995 for the Cochrane Statistical Methods Working Group

**Topic**	**Comments**
Continuous outcome measures	More work is needed on methods for combining results from trials with continuous outcome measures, especially with regard to the choice of effect measure, distribution of the data, baseline assessments and missing data. Of particular interest, perhaps, is the issue of combining results from trials where continuous outcome data have been categorised using varying cut-points and number of groups, or where some trials have presented results grouped and others as continuous.
Combining parallel group and cross-over trials	Work is needed to evaluate strategies for combining information from parallel group and cross-over trials, and to consider the information that needs to be supplied in reports of cross-over trials to enable them to be used in this way.
Heterogeneity	Given the lack of consensus at the workshop, it would be valuable to gain more insight into the merits of various strategies for combining trials when statistical heterogeneity is found. More empirical research into the effects of variation in methodology on heterogeneity would be valuable
Combining trials with different endpoints	It would be useful to consider what, if anything, can be done to combine trials that use different endpoints (This is a generalisation of the issue of combining data from trials where the same endpoint is assessed in different ways).
Summary statistics	More work is needed examining the relative merits of odds ratios, relative risks, NNTs and so on for meta-analysis of RCTs.
Extraction of summary statistics from published trials where the endpoint is survival time	Methods to combine results from survival studies where individual data are not available need to be investigated. Guidelines for minimum standards for reporting such trials should be developed.
Ignoring survival times	A study could be carried out of the loss of information (and power?) arising from classifying patients by presence or absence of the event of interest rather than by time to event. The effect of the varying length of follow-up should be examined.
Presentation of meta-analyses - numerical	Studies should be encouraged examining the merits of odds ratios, relative risks, NNT and other approaches for producing numerical summaries of the results of meta-analyses.
Presentation of meta-analyses - graphical	Various aspects of graphical presentation vary among published meta-analyses. The relative merits of these should be systematically reviewed. If possible, recommendations should be developed for standard graphical presentation. Aspects to consider include ordering of trials, symbols used, whether or not log scale used for treatment effect, and whether any additional graphs might usefully supplement the standard type.
Random effects models	It would be useful to have clarification of the properties of the various strategies for fitting random effects models.
Combining results from observational studies	Methods for combining results from observational studies, such as case-control studies, need to be investigated. In particular, methods are needed to pool estimated regression coefficients.

The participants at the 1993 meeting also noted the importance of studying the benefits of collecting individual patient data versus using published summary statistics. That topic was deemed to fit more within the remit of a specialist group, and a meeting of interested parties in Oxford in April 1994 led to the founding of the Meta-analyses using Individual Patient Data Methods Working Group [4].

In March 1995, an application was made for the “Cochrane Collaboration Working Group on Statistical Methods for Data Synthesis” to be formally registered. At that time the group put forward the following objectives:

• To develop and update guidelines on statistical methods for data synthesis.

• To develop and update guidelines for the integration of the methodological quality of randomised controlled trials into statistical methods for data synthesis.

• To serve as a “clearinghouse” for names of individuals who are willing and able to provide technical consultations to those in The Cochrane Collaboration on specific methodological issues.

It was recognised early that the SMG would benefit from funding to support methodological explorations and development of enhanced guidance, and the SMG made the following argument in an unsuccessful attempt to get funding in 1997:

“Many of the statistical queries and issues in the Cochrane Collaboration are not straightforward. While the basic statistical methods for meta-analysis are well known, in any particular systematic review it is unusual for these to be applicable without problems. For example there are often difficulties in extracting the required information from publications, in addressing both clinical and statistical heterogeneity between trials, in considering the potential effects of publication bias, in undertaking appropriate sensitivity analyses to investigate the robustness the conclusions made, and in investigating the effects of methodological quality of the primary studies on the overall results and interpretation. These difficult issues require both empirical and methodological work.”

In the absence of dedicated funding for the SMG, however, methodological advances by members of the group have been motivated by SMG discussions (and other Cochrane activities) but not determined by the SMG. Thus, progress addressing these research topics has been piecemeal. Yet members of the group have made many key contributions to advancing the statistical aspects of systematic review methods, as well as authoring several books on methods for systematic reviews and meta-analyses (for example, [5-9]). Thus evolution of statistical methodology used in Cochrane reviews was informed mainly through discussion of new developments initiated outside the Collaboration, although many SMG members were involved in those advances. In 1997, the SMG merged with the Quality of Reporting Trials Methods Group, led by David Moher, who became a co-convenor of the SMG.

Communication within SMG was always primarily via an email discussion list. Membership of the SMG grew (from 33 members in 1995, to 133 by 2006), with the group acting primarily as a discussion forum. To try to advance understanding of specific methodological areas in the late 1990s, the SMG established subgroups for specific research topics and members were invited to join these. The most overt success came from a group of SMG members who were interested in the incorporation of crossover trials in meta-analyses. Their work led to a journal publication [10].

Statistical issues relating to systematic review of diagnostic test accuracy were never part of the SMG’s remit, as the Screening and Diagnostic Tests Methods Group had also existed from the early years of the Collaboration. However, other parts of the SMG’s scope have split off into specialist Methods Groups over time. In 2000, the activities relating to problems of reporting research were taken up by a newly formed Reporting Bias Methods group (renamed the Bias Methods Group in 2005). Statistical methods for other specific contexts have also been addressed by the formation of other new methods groups, notably the Non-randomised Studies Methods Group in 1999 and Comparing Multiple Interventions Methods Group in 2010.

## The Statistical Methods Group and methodological developments in meta-analysis

The SMG has maintained and promoted a research agenda of issues important to the statistical synthesis of study findings. Important methodological developments have often been motivated through SMG members’ involvement in Cochrane reviews and through discussion on the SMG email list, at training events and during scientific meetings (see next section for more detail). Most of the research topics that were identified by the Group in 1995 (Table 1) have now been addressed, and the guidance based on this research is offered in the *Cochrane Handbook for Systematic Reviews of Interventions* (henceforth referred to as the *Cochrane Handbook*) [8]. The *Cochrane Handbook* has had substantial impact, with more than 7,000 citations, approximately 74% of which are from sources other than Cochrane Reviews [11]. While not all these citations can be attributed to the statistical chapters 9, 10 and 16 [12-14], these chapters are integral to the *Cochrane Handbook*, and are a key resource for statistical synthesis in systematic reviews.

The contribution of SMG members to the meta-analysis literature and the *Cochrane Handbook* has been extensive and has helped to shape the wider meta-analysis landscape. These contributions have included not only methodological developments, but also empirical studies that have evaluated existing methods. Below we highlight some landmark methodological developments that have influenced the field of meta-analysis. We include highly cited papers, and those that have received awards at Cochrane Colloquia (the annual conferences of The Cochrane Collaboration), made an important novel contribution, or generated important debate. This list is not exhaustive or fully representative of the research activity of SMG members. A full list of publications and current SMG members can be found online at smg.cochrane.org/publications and smg.cochrane.org/our-contributors.

The importance of the choice of the effect measure for dichotomous data in the presence of heterogeneity was examined by Deeks [15]. Results from this research have largely framed current guidance that suggests relative measures (odds ratio or risk ratio) to be preferable to absolute (risk difference) (for example, [9,12,16]). Sweeting *et al.*[17], Bradburn *et al.*[18] and others have evaluated the methodology for sparse dichotomous data and made suggestions for their optimal modelling [19,20]. The potential of the ratio of means for continuous outcomes as an alternative effect measure to the mean difference and standardised mean difference was presented at the 15^th^ and 16^th^ Cochrane Colloquia (in 2007 and 2008). A series of papers examining the performance of this measure have been published [21-23]. Different methods for meta-analysis of skewed data have been evaluated [24]. Missing outcome data in trials with dichotomous outcomes have been addressed in meta-analyses primarily through sensitivity analysis methods [25,26]. However, there are still many unanswered questions of how best to handle missing outcome data in meta-analysis of trials, particularly where the outcome is continuous, and this led The Cochrane Collaboration to fund a project on missing data in 2011. Methods for handling missing standard deviations in meta-analysis have been described and appraised in Wiebe *et al.*[27] and empirically evaluated [28]. Clinical fields where survival data are common have largely benefited from the development of methods to combine time-to-event data [29-31].

SMG members have made methodological contributions to the synthesis of results from trials with non-standard designs. Extensive research has been undertaken evaluating strategies for combining results from parallel group and cross-over trials [10,32-34], and simple methods to deal with paired data (such as eyes or arms) have been proposed [35]. These methods are recommended in the *Cochrane Handbook*.

The *I*^*2*^ statistic, which quantifies the amount of heterogeneity [36], is now probably the most popular method to evaluate heterogeneity (for example, [16,37,38]). The general medical journal article published in 2003 that describes *I*^*2*^ has been cited more than 6,000 times [39], and the statistic has been included in RevMan (The Cochrane Collaboration’s software to prepare and maintain reviews [40]). Problems with interpretation of the *I*^*2*^ statistic have been discussed [41]. Current guidance encourages reviewers to explore sources of heterogeneity by employing techniques such as subgroup analysis and meta-regression, although the pitfalls of such *post-hoc* analyses have been highlighted [42]. While meta-regression has not been implemented in RevMan, since this method is rarely appropriate in Cochrane reviews which typically include few studies [43], results and graphs from meta-regression fitted in statistical packages can be easily imported into RevMan. A significance test to investigate if there are differences between two or more subgroups has recently been added [9]. *Cochrane Handbook* guidance recommends the use of random effects in the presence of unexplained heterogeneity and the results are summarised in terms of an average combined effect size and its standard error. However, more recently, it has been proposed that in the presence of unexplained heterogeneity, an average effect is not informative, and that instead, the distribution of effects should be considered [44]. This has led to the development of different methods to calculate prediction intervals, which better convey heterogeneity of the results [44].

One particular source of heterogeneity that has been observed in meta-analyses is the difference in the magnitude of effects between small and large studies, often termed ‘small-study effects’. The *Cochrane Handbook* explicitly warns against misinterpreting funnel plot asymmetry as necessarily indicating publication bias, and the connections between heterogeneity, funnel plot asymmetry and small study effects have long been a focus of members of the SMG [45]. Consequently, SMG members have published several methodological developments evaluating the possibility of small study effects [46-52], visualizing them [53] and accounting for them [54-58].

SMG members have undertaken methodological work on network meta-analysis (also called multiple-treatments meta-analysis and mixed-treatment comparison), a multidimensional extension of meta-analysis aiming to make inferences about the relative effectiveness of many treatments for the same health condition. The methodology, presented in Higgins and Whitehead [59], takes advantage of indirect evidence in a network of interventions and has attracted much interest in recent years within the SMG and, more broadly, The Cochrane Collaboration. Workshops and contributing talks on the topic have been presented at Cochrane Colloquia since 2005. SMG members have contributed many methodological and applied publications on this topic in the scientific literature (see, for example, [60-69]).

The SMG recognised early on the potential of the method to answer policy-relevant questions and, because of the specialist nature of the methods, initiated the creation of the Comparing Multiple Interventions Methods Group (CMIMG). The role of the CMIMG is to develop and maintain guidance for undertaking and publishing Cochrane Reviews that compare multiple interventions via network meta-analysis. The promise of network meta-analysis has led to rapid development and adoption of the methods, stimulating collaboration between members of the CMIMG and researchers external to The Cochrane Collaboration.

Many of the SMG developments outlined above were initially presented at Cochrane Colloquia, and several have received the Thomas C Chalmers award, an award presented for best oral or poster presentation at a Cochrane Colloquium addressing methodological issues related to systematic reviews. SMG members have received in total 11 prizes on topics strictly within the scope of the SMG (Table 2), and several more for presentations that relate to bias, individual participant data and diagnostic tests.

**Table 2:** Thomas C Chalmers awards for statistical issues related to systematic reviews

**Year**	**Authors**	**Title**
1996	Liberati A, D’Amico R, Torri V, Tinazzi A, Leonetti C, Pifferi S	Meta-analyses from different sources of information
1998	Deeks J, Bradburn M, Bilker W, Localio R, Berlin J	Much ado about nothing: statistical methods for meta-analysis with rare events
1999	Higgins J	How should we interpret updated meta-analyses?
2001	Deeks JJ	Half dead or half alive? Which way should events be coded for meta-analyses of risk ratios?
2003	Hollis S and Preston C	Allowing for uncertainty due to missing data in a binary meta-analysis. Better than best/worst case analysis?
2005	Brok J, Thorlund K, Wetterslev J, Gluud C	Trial sequential analyses of six Cochrane neonatal group meta-analyses considering adequacy of allocation concealment
	Salanti G, Higgins J, Marinho V	How to determine the best treatment: a mixed-treatment-comparisons meta-analysis (MTM) of trials of topical fluoride therapies for the prevention of dental caries
2006	Skipka G	The inclusion of the estimated inter-study variation into forest plots for random-effects meta-analyses - a suggestion for a graphical presentation
2007	Friedrich J, Adhikari N, Ohlsson A, Beyene J	Ratio of means as an alternative to mean differences for analyzing continuous outcome variables in a meta-analysis: a simulation study
	Patsopoulos N, Ioannidis J, Evangelou E	Uncertainty of heterogeneity in meta-analysis
2008	Anzures-Cabrera J, Higgins JPT	Expressing meta-analyses of continuous outcomes in terms of risks

SMG members have also co-authored publications that fall under the remit of other Cochrane methods groups including the Individual Participant Data Meta-analysis Methods Group (ipdmamg.cochrane.org), Prospective Meta-Analysis Methods Group (pma.cochrane.org), Bias Methods Group (bmg.cochrane.org), Prognosis Methods Group (prognosismethods.cochrane.org) and the Non-Randomised Studies Methods Group.

## Implementation and dissemination of statistical methods within The Cochrane Collaboration

The SMG is involved in a range of activities within the Collaboration that aim to provide support to systematic review authors, the staff of Cochrane Review Groups and statisticians. A particular role of the SMG is to provide guidance when there are alternative approaches, such as handling multi-arm trials, handling missing outcomes within trials, imputing standard deviations when not presented, and mixing means with medians.

All CRGs are expected to involve statisticians [70]. They contribute to the CRGs in various ways including being statistical editors of CRGs; peer review, collaboration and authorship on systematic reviews; development of methods resources for CRGs; involvement in methodological quality improvement; and responding to statistical queries. In addition, statisticians in some CRGs have undertaken research to address methods issues prompted by their CRG (for example, [71-74]). The SMG maintains an email list that provides a forum for discussion of statistical issues relevant to systematic reviews. Membership of the list constitutes membership of the SMG, and includes CRG statisticians and other statisticians/methodologists with an interest in systematic review methods. The list currently has 237 members (April 2013), from 27 countries, primarily based in the UK (37%), USA (12%), Australia (10%), Germany (9%) and Canada (8%).

Members of the SMG contribute to the dissemination of statistical methods through training at regional Cochrane events and Colloquia, reviewing of standard review author training materials, and contributions to the *Cochrane Handbook*[8]. A set of core workshops, targeting review authors and CRG staff, are presented at Colloquia covering introductory topics on meta-analysis including: basic ideas, meta-analysis of binary and continuous outcomes, dealing with heterogeneity, and inclusion of non-standard studies and non-standard data. A full list of workshops presented at Colloquia is available at smg.cochrane.org/teachingtraining-workshops. The SMG has run two major training events for Cochrane statisticians. The first ‘Summer school for Cochrane Statisticians’ was held in Oxford, UK, in July 2001, while the second, ‘Cochrane Statistical Methods Group Training Course: Addressing advanced issues in meta-analytical technique’ was held in Cardiff, UK, in April 2010. The latter course led to a series of freely available online materials, including slidecasts of the presentations (smg.cochrane.org/teachingtraining-workshops/smg_training_course_2010).

In addition to training workshops, SMG members have organised and presented in SMG scientific meetings, special methods sessions, and plenaries at Cochrane Colloquia. The purpose of these sessions has often been to discuss or present new or controversial issues in meta-analysis. *Ad hoc* meetings outside Cochrane Colloquia have also been convened to discuss specific methodological issues. Examples of key events are presented in Table 3.

**Table 3:** Description of the Statistical Methods Group scientific meetings, special methods sessions, and plenaries

**Year**	**Event**	**Description of event**
1993	Oxford Colloquium	Statistical Methods Working Group (later SMG) scientific meeting. Discussed the range of statistical methods that The Cochrane Collaboration could use in undertaking meta-analyses and noted a lack of consensus in appropriate methods.
1996	Oxford	1^st^*Ad hoc* meeting. Discussed an eclectic set of issues relating to the development of the MetaView software (later developed into RevMan) and various methodological issues.
1996	Oxford	2^nd^*Ad hoc* meeting. Convened to discuss issues relevant to the analysis of ordinal data.
1998	Oxford	3^rd^*Ad hoc* meeting. Discussed issues related to cluster randomised trials in systematic reviews.
2001	Lyon Colloquium	SMG scientific meeting. Discussion on fixed versus random effects meta-analysis and intention-to-treat analysis. Consensus decision made regarding heterogeneity: that a test for heterogeneity should not be used to decide between fixed and random effects models.
2002	Oxford Colloquium	SMG scientific meeting: Several methodological issues discussed: cross-over trials, combining studies with different designs and methods for dealing with heterogeneity.
2004	Edinburgh	4^th^*Ad hoc* meeting. Discussed issues relating to the quality assessment of trials.
2006	Melbourne Colloquium	Special methods session: “Assessing susceptibility to bias of included studies: new recommendations for Cochrane reviews”. First open meeting to introduce and discuss the proposed Risk of Bias tool (as was later named).
2008	Freiburg Colloquium	Satellite event to the Freiburg Colloquium: Joint meeting of the SMG and the UK Meta-analysis in Medicine Group. Four sessions on: meta-analysis of continuous data, various topics, addressing risk of bias, and a debate “When should meta-analyses be performed in Cochrane reviews?”
		Special methods session “Some awkward statistical issues in Cochrane reviews”. Two topics “Subsets of studies in meta-analysis” and “Meta-analysis with multiple treatment groups”.
2009	Singapore Colloquium	Special methods session. Two parts: Part 1 “Exploring new approaches for dealing with bias and heterogeneity” and Part 2 “RevMan and beyond for meta-analysis”.
		Joint scientific meeting of the SMG and the Bias Methods Group. Three presentations on the assessment and impact of outcome reporting bias on systematic reviews, searching for methods studies, and a decision tool for updating Cochrane reviews.
2011	Madrid Colloquium	SMG scientific meeting. Two topics, multivariate meta-analysis and trial sequential analysis, were presented and discussed.
2012	Auckland Colloquium	Methods plenary. Theme of plenary was “Beyond healthcare decisions: systematic reviews as a tool for informing future research and research methodology”.
2013	Québec Colloquium	Satellite event to the Québec City Colloquium: “Data, Outcomes, Uncertainty and Graphs: Advances and Limitations in Trials, Meta-Analysis, and Novelties”. Four sessions on: selective reporting, bias, statistical issues, and presentations considering the case for and against inclusion of funding source as a standard item in the Cochrane Risk of Bias tool.

## Discussion

### What has statistics done for The Cochrane Collaboration?

The need for statistical synthesis methods to enable interpretation of results from multiple studies was recognised more than 50 years ago [1,75-77]. Meta-analyses now form a core component of many Cochrane Reviews. Without such a method, interpreting the effectiveness of an intervention is difficult, if not impossible. Further, meta-analysis allows the combining of results from a series of small studies, to answer a question regarding the effectiveness of an intervention, which could otherwise not be answered from the individual studies. Extensions of meta-analysis methods provide systematic review authors with an extensive tool kit to answer questions beyond whether an intervention is effective. For example, which factors may modify the magnitude of the intervention effect [42], what is the likely effect of an intervention in an individual setting [44], or what is the relative effectiveness of many interventions for the same health condition [64]. These extensions increase the utility of systematic reviews in answering key questions of relevance to policy makers, health care decision makers and patients.

### What has The Cochrane Collaboration done for statistics?

The Cochrane Collaboration has provided the impetus, structure and coordination for an international group of methodologists to contribute to the development of statistical aspects of systematic review methods. This has expedited development of systematic review methods that would not likely have been achieved as rapidly without such an organisation. Coupled with the benefits of an international collaboration of methodologists, the main output of The Cochrane Collaboration, the *Cochrane Database of Systematic Reviews*, has facilitated methodological research, by providing a large and unique repository of data on systematic reviews and meta-analyses (for example, [23,43,78-83]). A recent paper highlighted those articles published in statistical journals that appear to be having the most direct impact on general and internal medicine research [84]. The authors identified the 18 most-cited biostatistical articles; 11 were about methodological aspects of meta-analysis, 7 of which were co-authored by SMG members [17,18,32,36,42,48,64].

### What are the research gaps in meta-analysis methods?

Further research to establish the optimal strategy for combining intervention effects estimated from analyses of final values and change scores is required [85]; SMG members are currently investigating this area [86]. Further research is also needed to develop and evaluate methods for combining results from trials where continuous outcome data have been categorised using varying cut-points [87]. A related issue is the best approach to deal with the scenario where trials present results for the same outcome based on a mixture of continuous and dichotomous measures. Statistical methods that adjust for potential bias in included studies (as, for example, in Turner *et al.*[57]) might be desirable, particularly when non-randomised studies are included. Evaluation of methods to adjust for ‘hidden’ clustering in individually randomised trials arising through the organisation of the intervention (for example, therapists treating groups of patients, or group therapy sessions, surgical procedures) would be useful [88,89]. Development of methodology for meta-analysis of time-to-event data that allow for more complex multistate modeling, such as competing risks, is needed. Several developments have taken place in recent years in the field of multivariate meta-analysis [90-92]. The potential of different methods (for example, maximum likelihood, method of moments and Bayesian approaches) to estimate parameters of multivariate meta-analysis models and the estimation of within-study correlation in the absence of detailed study-level data needs further attention [93,94]. Methods to describe and quantify heterogeneity (for example, extending the *I*^*2*^ statistic) and visually display results from multivariate meta-analysis have been recently suggested [95] and their usefulness and performance remains to be tested in practice. Finally, the appropriateness of the statistical methods used in Cochrane Reviews requires regular assessment, particularly as new methods appear in the literature. Cochrane reviews have specific characteristics (for example, they typically have few studies [43]) and knowledge of how synthesis methods perform, in which circumstances their performance is most compromised, and how large the impact is when this occurs, is required to develop guidance for the *Cochrane Handbook*, and include the most appropriate methods in RevMan. Simulation studies and extensive meta-epidemiological studies provide the necessary research to underpin this guidance.

### Thoughts on our future direction

While 10 years ago, say, some may have felt that all the main statistical issues in systematic reviews had been addressed, the reality is that new challenges have continued to arise, and the pace of methodological work has not slowed. While we cannot say in which areas future innovations will appear we are confident that such innovations will indeed be made. And we expect the SMG members to continue to be among those at the forefront of this work. The SMG will continue to have a key role in advising The Cochrane Collaboration on which methods are, or are not, recommended, or whether new approaches need more evaluation before a decision can be made. They also have an important role in further refinement and extension of the handbook and other training materials.

Despite the contribution SMG members have made to advance the statistical aspects of systematic review methods, funding the work of the SMG and, more broadly, methodological research in evidence synthesis remains an ongoing challenge. Methodological research funding programmes, such as those provided in the United Kingdom (for example, the Medical Research Council/National Institute for Health Research Methodology Research Programme), United States (for example, Agency for Healthcare Research and Quality research grants for comparative effectiveness), and Europe (for example, the European Commission’s Seventh Framework Programme and Horizon 2020), are critical for the ongoing development and evaluation of methods, and production of guidance. Specific funding for the SMG would allow for a more priority-driven and structured approach to addressing important gaps in statistical synthesis methods that are of particular relevance to Cochrane Reviews, thus expediting the availability of research on which to base well-informed decisions. Incorporation into the Cochrane Handbook and RevMan of new statistical synthesis methods that make the best use of evidence will contribute to The Cochrane Collaboration’s goal of producing high quality research evidence. Given the impact of the Cochrane Handbook and the widespread use of RevMan, the benefits would likely extend well beyond reviews completed under the auspices of The Cochrane Collaboration.

## Conclusions

Over the past 20 years, the SMG has played a pivotal role within The Cochrane Collaboration in determining the direction of statistical methods used within Cochrane reviews. Members of the SMG have made key contributions to advancing statistical aspects of systematic reviews. Many research gaps in statistical methods of systematic reviews remain, and will continue to arise as the field of meta-analysis develops. Statisticians and methodologists will lead the development and evaluation of these methods, thus being critical players in the production of evidence.

## Competing interests

All authors are members of the SMG. JEM and GS are current co-convenors and SCL and DGA are past co-convenors of the SMG.

## Authors’ contributions

SCL (past convenor of the SMG) and JEM (current convenor of the SMG) planned the paper. SCL drafted an earlier manuscript that informed the content of the current manuscript. GS (current convenor), DGA (convenor up to September 2013), and JEM wrote the first draft of this manuscript. All authors contributed to revisions of the manuscript, and read and approved the final manuscript.

## Supplementary Material

Additional file 1 Oxford 1993 Workshop Report. Click here for file

## References

[B1] Glass GV Primary, secondary, and meta-analysis of research Educ Res 1976 5 3 8

[B2] Chalmers I Hetherington J Elbourne D Keirse MJNC Enkin M Chalmers I, Enkin M, Keirse MJNC Materials and methods used in synthesizing evidence to evaluate the effects of care during pregnancy and childbirth Effective Care in Pregnancy and Childbirth 1989 Oxford; New York: Oxford Medical Publications 39 65

[B3] Chalmers I Altman DG Systematic Reviews 1995 London: BMJ Publishing Group

[B4] Stewart LA Clarke MJ Practical methodology of meta-analyses (overviews) using updated individual patient data. Cochrane Working Group Stat Med 1995 14 2057 2079 10.1002/sim.4780141902 8552887

[B5] Sutton AJ Abrams KR Jones DR Sheldon TA Song F Methods for Meta-analysis in Medical Research 2000 Chinchester: John Wiley and Sons Ltd

[B6] Egger M Davey Smith G Altman DG Systematic Reviews in Health Care: Meta-Analysis in Context 2001 2 London: BMJ Publishing Group

[B7] Whitehead A Meta-Analysis of Controlled Clinical Trials 2003 Chinchester, UK: John Wiley & Sons, Ltd

[B8] Higgins JPT Green S Cochrane Collaboration Cochrane Handbook for Systematic Reviews of Interventions 2008 Chichester, England. Hoboken, NJ: Wiley-Blackwell

[B9] Borenstein M Hedges LV Higgins J Rothstein H Introduction to Meta-Analysis 2009 Chichester, UK: John Wiley & Sons Inc.

[B10] Elbourne DR Altman DG Higgins JP Curtin F Worthington HV Vail A Meta-analyses involving cross-over trials: methodological issues Int J Epidemiol 2002 31 140 149 10.1093/ije/31.1.140 11914310

[B11] Google Scholarhttp://scholar.google.com.au/scholar?as_vis=0&hl=en&as_sdt=2005&sciodt=0,5&cites=15756404854880902392&scipsc = (date accessed: 20th August 2013)

[B12] Deeks JJ Higgins JPT Altman DG Higgins JPT, Green S Chapter 9: Analysing data and undertaking meta-analyses Cochrane Handbook for Systematic Reviews of Interventions 2008 Chichester, UK: John Wiley & Sons, Inc 243 296

[B13] Sterne JA Egger M Moher D Higgins JPT, Green S Chapter 10: Addressing reporting biases Cochrane Handbook for Systematic Reviews of Interventions 2008 Chichester, UK: John Wiley & Sons, Inc 297 334

[B14] Higgins JPT Deeks JJ Altman DG Higgins JPT, Green S Chapter 16: Special topics in statistics Cochrane Handbook for Systematic Reviews of Interventions 2008 Chichester, UK: John Wiley & Sons, Inc 481 530

[B15] Deeks JJ Issues in the selection of a summary statistic for meta-analysis of clinical trials with binary outcomes Stat Med 2002 21 1575 1600 10.1002/sim.1188 12111921

[B16] Centre for Reviews and Dissemination Systematic Reviews: CRD’s Guidance for Undertaking Reviews in Health Care 2008 York: CRD, University of York

[B17] Sweeting MJ Sutton AJ Lambert PC What to add to nothing? Use and avoidance of continuity corrections in meta-analysis of sparse data Stat Med 2004 23 1351 1375 10.1002/sim.1761 15116347

[B18] Bradburn MJ Deeks JJ Berlin JA Russell Localio A Much ado about nothing: a comparison of the performance of meta-analytical methods with rare events Stat Med 2007 26 53 77 10.1002/sim.2528 16596572

[B19] Friedrich JO Adhikari NK Beyene J Inclusion of zero total event trials in meta-analyses maintains analytic consistency and incorporates all available data BMC Med Res Methodol 2007 7 5 10.1186/1471-2288-7-5 17244367 PMC1783664

[B20] Rucker G Schwarzer G Carpenter J Olkin I Why add anything to nothing? The arcsine difference as a measure of treatment effect in meta-analysis with zero cells Stat Med 2009 28 721 738 10.1002/sim.3511 19072749

[B21] Friedrich JO Adhikari NK Beyene J The ratio of means method as an alternative to mean differences for analyzing continuous outcome variables in meta-analysis: a simulation study BMC Med Res Methodol 2008 8 32 10.1186/1471-2288-8-32 18492289 PMC2430201

[B22] Friedrich JO Adhikari NK Beyene J Ratio of means for analyzing continuous outcomes in meta-analysis performed as well as mean difference methods J Clin Epidemiol 2011 64 556 564 10.1016/j.jclinepi.2010.09.016 21447428

[B23] Friedrich JO Adhikari NK Beyene J Ratio of geometric means to analyze continuous outcomes in meta-analysis: comparison to mean differences and ratio of arithmetic means using empiric data and simulation Stat Med 2012 31 1857 1886 10.1002/sim.4501 22438170

[B24] Higgins JP White IR Anzures-Cabrera J Meta-analysis of skewed data: combining results reported on log-transformed or raw scales Stat Med 2008 27 6072 6092 10.1002/sim.3427 18800342 PMC2978323

[B25] Gamble C Hollis S Uncertainty method improved on best-worst case analysis in a binary meta-analysis J Clin Epidemiol 2005 58 579 588 10.1016/j.jclinepi.2004.09.013 15878471

[B26] Higgins JP White IR Wood AM Imputation methods for missing outcome data in meta-analysis of clinical trials Clin Trials 2008 5 225 239 10.1177/1740774508091600 18559412 PMC2602608

[B27] Wiebe N Vandermeer B Platt RW Klassen TP Moher D Barrowman NJ A systematic review identifies a lack of standardization in methods for handling missing variance data J Clin Epidemiol 2006 59 342 353 10.1016/j.jclinepi.2005.08.017 16549255

[B28] Thiessen Philbrook H Barrowman N Garg AX Imputing variance estimates do not alter the conclusions of a meta-analysis with continuous outcomes: a case study of changes in renal function after living kidney donation J Clin Epidemiol 2007 60 228 240 10.1016/j.jclinepi.2006.06.018 17292016

[B29] Parmar MK Torri V Stewart L Extracting summary statistics to perform meta-analyses of the published literature for survival endpoints Stat Med 1998 17 2815 2834 10.1002/(SICI)1097-0258(19981230)17:24<2815::AID-SIM110>3.0.CO;2-8 9921604

[B30] Williamson PR Smith CT Hutton JL Marson AG Aggregate data meta-analysis with time-to-event outcomes Stat Med 2002 21 3337 3351 10.1002/sim.1303 12407676

[B31] Tierney JF Stewart LA Ghersi D Burdett S Sydes MR Practical methods for incorporating summary time-to-event data into meta-analysis Trials 2007 8 16 10.1186/1745-6215-8-16 17555582 PMC1920534

[B32] Curtin F Altman DG Elbourne D Meta-analysis combining parallel and cross-over clinical trials. I: Continuous outcomes Stat Med 2002 21 2131 2144 10.1002/sim.1205 12210629

[B33] Curtin F Elbourne D Altman DG Meta-analysis combining parallel and cross-over clinical trials. II: Binary outcomes Stat Med 2002 21 2145 2159 10.1002/sim.1206 12210630

[B34] Curtin F Elbourne D Altman DG Meta-analysis combining parallel and cross-over clinical trials. III: The issue of carry-over Stat Med 2002 21 2161 2173 10.1002/sim.1207 12210631

[B35] Perera R Glasziou P A simple method to correct for the design effect in systematic reviews of trials using paired dichotomous data J Clin Epidemiol 2007 60 975 978 10.1016/j.jclinepi.2006.12.004 17689815

[B36] Higgins JP Thompson SG Quantifying heterogeneity in a meta-analysis Stat Med 2002 21 1539 1558 10.1002/sim.1186 12111919

[B37] Fu R Gartlehner G Grant M Shamliyan T Sedrakyan A Wilt TJ Griffith L Oremus M Raina P Ismaila A Santaguida P Lau J Trikalinos TA Conducting quantitative synthesis when comparing medical interventions: AHRQ and the Effective Health Care Program J Clin Epidemiol 2011 64 1187 1197 10.1016/j.jclinepi.2010.08.010 21477993

[B38] Guyatt GH Oxman AD Kunz R Woodcock J Brozek J Helfand M Alonso-Coello P Glasziou P Jaeschke R Akl EA Norris S Vist G Dahm P Shukla VK Higgins J Falck-Ytter Y Schünemann HJ GRADE Working Group GRADE guidelines: 7. Rating the quality of evidence - inconsistency J Clin Epidemiol 2011 64 1294 1302 10.1016/j.jclinepi.2011.03.017 21803546

[B39] Higgins JP Thompson SG Deeks JJ Altman DG Measuring inconsistency in meta-analyses BMJ 2003 327 557 560 10.1136/bmj.327.7414.557 12958120 PMC192859

[B40] The Nordic Cochrane Centre, Copenhagen Review Manager (RevMan)[Computer program]. Version 5.2 2012 The Cochrane Collaboration

[B41] Rucker G Schwarzer G Carpenter JR Schumacher M Undue reliance on I(2) in assessing heterogeneity may mislead BMC Med Res Methodol 2008 8 79 10.1186/1471-2288-8-79 19036172 PMC2648991

[B42] Thompson SG Higgins JP How should meta-regression analyses be undertaken and interpreted? Stat Med 2002 21 1559 1573 10.1002/sim.1187 12111920

[B43] Davey J Turner RM Clarke MJ Higgins JP Characteristics of meta-analyses and their component studies in the Cochrane Database of Systematic Reviews: a cross-sectional, descriptive analysis BMC Med Res Methodol 2011 11 160 10.1186/1471-2288-11-160 22114982 PMC3247075

[B44] Higgins JP Thompson SG Spiegelhalter DJ A re-evaluation of random-effects meta-analysis J R Stat Soc Ser A Stat Soc 2009 172 137 159 10.1111/j.1467-985X.2008.00552.x 19381330 PMC2667312

[B45] Sterne JA Sutton AJ Ioannidis JP Terrin N Jones DR Lau J Carpenter J Rucker G Harbord RM Schmid CH Tetzlaff J Deeks JJ Peters J Macaskill P Schwarzer G Duval S Altman DG Moher D Higgins JP Recommendations for examining and interpreting funnel plot asymmetry in meta-analyses of randomised controlled trials BMJ 2011 343 d4002 10.1136/bmj.d4002 21784880

[B46] Sterne JA Gavaghan D Egger M Publication and related bias in meta-analysis: power of statistical tests and prevalence in the literature J Clin Epidemiol 2000 53 1119 1129 10.1016/S0895-4356(00)00242-0 11106885

[B47] Sterne JA Egger M Smith GD Systematic reviews in health care: investigating and dealing with publication and other biases in meta-analysis BMJ 2001 323 101 105 10.1136/bmj.323.7304.101 11451790 PMC1120714

[B48] Macaskill P Walter SD Irwig L A comparison of methods to detect publication bias in meta-analysis Stat Med 2001 20 641 654 10.1002/sim.698 11223905

[B49] Harbord RM Egger M Sterne JA A modified test for small-study effects in meta-analyses of controlled trials with binary endpoints Stat Med 2006 25 3443 3457 10.1002/sim.2380 16345038

[B50] Schwarzer G Antes G Schumacher M A test for publication bias in meta-analysis with sparse binary data Stat Med 2007 26 721 733 10.1002/sim.2588 16755545

[B51] Rucker G Schwarzer G Carpenter J Arcsine test for publication bias in meta-analyses with binary outcomes Stat Med 2008 27 746 763 10.1002/sim.2971 17592831

[B52] Carpenter JR Schwarzer G Rucker G Kunstler R Empirical evaluation showed that the Copas selection model provided a useful summary in 80% of meta-analyses J Clin Epidemiol 2009 62 624 631 10.1016/j.jclinepi.2008.12.002 19282148

[B53] Peters JL Sutton AJ Jones DR Abrams KR Rushton L Contour-enhanced meta-analysis funnel plots help distinguish publication bias from other causes of asymmetry J Clin Epidemiol 2008 61 991 996 10.1016/j.jclinepi.2007.11.010 18538991

[B54] Riley RD Sutton AJ Abrams KR Lambert PC Sensitivity analyses allowed more appropriate and reliable meta-analysis conclusions for multiple outcomes when missing data was present J Clin Epidemiol 2004 57 911 924 10.1016/j.jclinepi.2004.01.018 15504634

[B55] Peters JL Sutton AJ Jones DR Abrams KR Rushton L Performance of the trim and fill method in the presence of publication bias and between-study heterogeneity Stat Med 2007 26 4544 4562 10.1002/sim.2889 17476644

[B56] Moreno SG Sutton AJ Ades AE Stanley TD Abrams KR Peters JL Cooper NJ Assessment of regression-based methods to adjust for publication bias through a comprehensive simulation study BMC Med Res Methodol 2009 9 2 10.1186/1471-2288-9-2 19138428 PMC2649158

[B57] Turner RM Spiegelhalter DJ Smith GC Thompson SG Bias modelling in evidence synthesis J R Stat Soc Ser A Stat Soc 2009 172 21 47 10.1111/j.1467-985X.2008.00547.x 19381328 PMC2667303

[B58] Rucker G Carpenter JR Schwarzer G Detecting and adjusting for small-study effects in meta-analysis Biom J 2011 53 351 368 10.1002/bimj.201000151 21374698

[B59] Higgins JP Whitehead A Borrowing strength from external trials in a meta-analysis Stat Med 1996 15 2733 2749 10.1002/(SICI)1097-0258(19961230)15:24<2733::AID-SIM562>3.0.CO;2-0 8981683

[B60] Caldwell DM Ades AE Higgins JP Simultaneous comparison of multiple treatments: combining direct and indirect evidence BMJ 2005 331 897 900 10.1136/bmj.331.7521.897 16223826 PMC1255806

[B61] Glenny AM Altman DG Song F Sakarovitch C Deeks JJ D’Amico R Bradburn M Eastwood AJ Indirect comparisons of competing interventions Health Technol Assess 2005 9 1 134 16014203 10.3310/hta9260

[B62] Caldwell DM Welton NJ Ades AE Mixed treatment comparison analysis provides internally coherent treatment effect estimates based on overviews of reviews and can reveal inconsistency J Clin Epidemiol 2010 63 875 882 10.1016/j.jclinepi.2009.08.025 20080027

[B63] Sutton A Ades AE Cooper N Abrams K Use of indirect and mixed treatment comparisons for technology assessment Pharmacoeconomics 2008 26 753 767 10.2165/00019053-200826090-00006 18767896

[B64] Salanti G Higgins JP Ades AE Ioannidis JP Evaluation of networks of randomized trials Stat Methods Med Res 2008 17 279 301 17925316 10.1177/0962280207080643

[B65] Song F Loke YK Walsh T Glenny AM Eastwood AJ Altman DG Methodological problems in the use of indirect comparisons for evaluating healthcare interventions: survey of published systematic reviews BMJ 2009 3 b1147 19346285 10.1136/bmj.b1147 PMC2665205

[B66] Dias S Welton NJ Caldwell DM Ades AE Checking consistency in mixed treatment comparison meta-analysis Stat Med 2010 29 932 944 10.1002/sim.3767 20213715

[B67] Li T Puhan MA Vedula SS Singh S Dickersin K Ad Hoc Network Meta-analysis Methods Meeting Working Group Network meta-analysis-highly attractive but more methodological research is needed BMC Med 2011 9 79 10.1186/1741-7015-9-79 21707969 PMC3159133

[B68] Mills EJ Ioannidis JP Thorlund K Schunemann HJ Puhan MA Guyatt GH How to use an article reporting a multiple treatment comparison meta-analysis JAMA 2012 308 1246 1253 10.1001/2012.jama.11228 23011714

[B69] Salanti G Indirect and mixed-treatment comparison, network, or multiple-treatments meta-analysis: many names, many benefits, many concerns for the next generation evidence synthesis tool Res Synth Methods 2012 3 80 97 10.1002/jrsm.1037 26062083

[B70] The Cochrane Collaboration The Cochrane Policy Manual [updated 7 May 2013] [http://www.cochrane.org/policy-manual/welcome]

[B71] Jones AP Remmington T Williamson PR Ashby D Smyth RL High prevalence but low impact of data extraction and reporting errors were found in Cochrane systematic reviews J Clin Epidemiol 2005 58 741 742 10.1016/j.jclinepi.2004.11.024 15939227

[B72] Riley RD Gates S Neilson J Alfirevic Z Statistical methods can be improved within Cochrane pregnancy and childbirth reviews J Clin Epidemiol 2011 64 608 618 10.1016/j.jclinepi.2010.08.002 21109399

[B73] Weir CJ Brady M Lewis SC Murray GD Langhorne P Practical Methods for Meta-Analysis of Continuous Outcomes in Stroke Rehabilitation Trials 2013 Funded grant from the United Kingdom Stroke Association

[B74] Weir J Lewis S Sandercock P Thomas B Murray G Meta-analysis of ordinal outcome measures in stroke trials. Can we do better? (P3B367) Cochrane Database Syst Rev 2011 3 233

[B75] Yates F Cochran WG The analysis of groups of experiments J Agric Sci 1938 28 556 580 10.1017/S0021859600050978

[B76] Chalmers I Hedges LV Cooper H A brief history of research synthesis Eval Health Prof 2002 25 12 37 10.1177/0163278702025001003 11868442

[B77] O’Rourke K An historical perspective on meta-analysis: dealing quantitatively with varying study results J R Soc Med 2007 100 579 582 10.1258/jrsm.100.12.579 18065712 PMC2121629

[B78] Dechartres A Trinquart L Boutron I Ravaud P Influence of trial sample size on treatment effect estimates: meta-epidemiological study BMJ 2013 346 f2304 10.1136/bmj.f2304 23616031 PMC3634626

[B79] Herbison P Hay-Smith J Gillespie WJ Adjustment of meta-analyses on the basis of quality scores should be abandoned J Clin Epidemiol 2006 59 1249 1256 17098567 10.1016/j.jclinepi.2006.03.008

[B80] Pattanittum P Laopaiboon M Moher D Lumbiganon P Ngamjarus C A comparison of statistical methods for identifying out-of-date systematic reviews PloS One 2012 7 e48894 10.1371/journal.pone.0048894 23185281 PMC3502410

[B81] Pereira TV Horwitz RI Ioannidis JP Empirical evaluation of very large treatment effects of medical interventions JAMA 2012 308 1676 1684 10.1001/jama.2012.13444 23093165

[B82] Turner RM Davey J Clarke MJ Thompson SG Higgins JP Predicting the extent of heterogeneity in meta-analysis, using empirical data from the Cochrane Database of Systematic Reviews Int J Epidemiol 2012 41 818 827 10.1093/ije/dys041 22461129 PMC3396310

[B83] Thorlund K Wetterslev J Awad T Thabane L Gluud C Comparison of statistical inferences from the DerSimonian-Laird and alternative random-effects model meta-analyses - an empirical assessment of 920 Cochrane primary outcome meta-analyses Res Synth Methods 2012 2 238 253 10.1002/jrsm.53 26061888

[B84] Nietert PJ Wahlquist AE Herbert TL Characteristics of recent biostatistical methods adopted by researchers publishing in general/internal medicine journals Stat Med 2013 32 1 10 10.1002/sim.5311 22415768 PMC3521084

[B85] Abrams KR Gillies CL Lambert PC Meta-analysis of heterogeneously reported trials assessing change from baseline Stat Med 2005 24 3823 3844 10.1002/sim.2423 16320285

[B86] McKenzie JE Methodological issues in meta-analysis of randomised controlled trials with continuous outcomes Doctor of Philosophy, Monash University, School of Public Health and Preventive Medicine 2011

[B87] Anzures-Cabrera J Sarpatwari A Higgins JP Expressing findings from meta-analyses of continuous outcomes in terms of risks Stat Med 2011 30 2967 2985 10.1002/sim.4298 21826697

[B88] Lee KJ Thompson SG Clustering by health professional in individually randomised trials BMJ 2005 330 142 144 10.1136/bmj.330.7483.142 15649931 PMC544437

[B89] Lee KJ Thompson SG The use of random effects models to allow for clustering in individually randomized trials Clin Trials 2005 2 163 173 10.1191/1740774505cn082oa 16279138

[B90] Jackson D Riley RD A refined method for multivariate meta-analysis and meta-regression Stat Med 2013 [Epub ahead of print] 10.1002/sim.5957 PMC4285306 23996351

[B91] Jackson D White IR Riley RD A matrix-based method of moments for fitting the multivariate random effects model for meta-analysis and meta-regression Biom J 2013 55 231 245 10.1002/bimj.201200152 23401213 PMC3806037

[B92] Jackson D White IR Thompson SG Extending DerSimonian and Laird’s methodology to perform multivariate random effects meta-analyses Stat Med 2010 29 1282 1297 19408255 10.1002/sim.3602

[B93] Jackson D Riley R White IR Multivariate meta-analysis: potential and promise Stat Med 2011 30 2481 2498 10.1002/sim.4172 PMC3470931 21268052

[B94] Mavridis D Salanti G A practical introduction to multivariate meta-analysis Stat Methods Med Res 2013 22 133 158 10.1177/0962280211432219 22275379

[B95] Jackson D White IR Riley RD Quantifying the impact of between-study heterogeneity in multivariate meta-analyses Stat Med 2012 31 3805 3820 10.1002/sim.5453 22763950 PMC3546377

