# Supplementary material for: Meta-analysis and The Cochrane Collaboration: 20 years of the Cochrane Statistical Methods Group
Source: Syst Rev. 2013 Nov 26;2:80. doi: 10.1186/2046-4053-2-80 (PMC4219183; doi:10.1186/2046-4053-2-80)
Supplement: Additional file 1 — Oxford 1993 Workshop Report. [file 2046-4053-2-80-S1.pdf]

# Statistical Methods for Data Synthesis:

## Cochrane Collaboration Workshop Report

## Introduction

The Cochrane Collaboration has evolved in response to Archie Cochrane's criticism of the medical profession for not having organised a critical summary, by specialty or subspecialty, adapted periodically, of all relevant randomised controlled trials. The Collaboration is developing the pilot edition of a 'Tool Kit' to inform, help and guide individuals and groups contributing systematic overviews (i.e. reviews with respect to scientific principles, in particular, the control of biases and random errors) to the Collaboration. This workshop was convened to develop guidelines on statistical methods for data synthesis for incorporation into the Tool Kit, and to identify useful research topics in this arena.

On July 16, 1993, 16 participants met at the UK Cochrane Centre. All were statisticians, epidemiologists, or physicians with expertise in statistical methods for data synthesis. Two separate sessions took place: the first on **Effect Measures** chaired by Stuart Pocock, and the second on **Approaches for Aggregation** chaired by Peter Armitage.

We had intended the deliberations to assume that only published summary statistics would be available since most overviews for the foreseeable future will continue to depend upon grouped data. Although most of the discussions did assume this, the preferability of having individual participant data was emphasized on a number of occasions. We did not

address issues on inclusion criteria for systematic overviews except for those bearing on methodological quality. Although we had also not intended to address issues relating to the presentation of results, separate consideration of methods and presentation proved impossible. Hence, we have provided some recommendations on presentation.

The recommendations in this report are based upon our impressions of the deliberations, upon the results of a detailed, self-administered questionnaire developed explicitly for the workshop, and upon comments received from participants on an earlier draft. Greater weight was given to the questionnaire since it provides evidence that is more objective and is documented. Fourteen of the 16 participants returned completed questionnaire forms.

## Effect Measures

Opening the meeting, Andy Oxman described the need for the 'Tool Kit' to contain guidelines for reviewers reflecting areas of reasonable consensus, and identifying those on which considerable disagreement prevailed. His view that it was desirable for the statistical methods to be consistent across reviews, as far as possible, was endorsed by most participants (10/14). He also suggested that the software should have explicit default methods of analysis and presentation, but that it should be possible for reviewers to override these. All members of the group supported this principle to some degree.

Most of the discussion in this session related to which effect measures should be used as

the default and which others might be available. Greatest emphasis was given to studies with binary outcome measures. The relative merits of the odds ratio and relative risk were discussed at length, especially in relation to the frequency of the outcome (event rate).

While it is well known that the odds ratio is a poor approximation to the relative risk when the event rate is high, it was pointed out that the use of the relative risk is also problematic in such cases. However effective the treatment, the upper bound of the relative risk is constrained. Thus when combining results from trials with a wide range of event rates, the odds ratio is likely to be more homogeneous across studies.

The odds ratio had the most overall support, with almost all choosing it as their method of choice when the event rate was typically lower than 20% and 5/14 choosing it regardless of the event rate. Some of the lack of wholehearted support was probably related to the feeling that the odds ratio was not the best summary for communicating results to doctors (see later). Rather less discussion was devoted to the risk difference. The relative risk and the risk difference received minimal support from participants as a preferred outcome measure, even for high event rates. Nevertheless, nine participants felt that the software should include all of the odds ratio (OR), relative risk (RR) and risk difference (RD) as options for the effect measure, with four supporting OR and RR only and one choosing OR and RD. Thus the clear message was that the Tool Kit default should be the OR, with the RR and probably also the RD as options.

Ten participants supported the idea that it would be useful to analyse the data using

alternative outcome measures if there was any ambiguity over which was the most appropriate, while four disagreed with this suggestion. Four participants felt that the choice of effect measure should depend in part on a test of heterogeneity, while the other ten disagreed.

Several participants felt that it would be preferable to use different measures for presentation than were used for analysing the data; this issue is addressed later.

Studies with continuous outcome measures have received much less attention in medical research than in areas such as education. Mike Bracken presented an approach analogous to that used for binary endpoints, in which mean differences from each study are combined using weights based on their variances (Bracken, 1992). Participants found little difficulty with this approach, but other approaches were mentioned, such as expressing the mean difference as a multiple of the control group standard deviation ('effect size'). Although three participants felt that there were no particular problems with overviews of such studies, most felt that the area merited deeper study. Issues raised included the choice of effect measure, the issue of data distribution (and whether using medians might be more appropriate than using means), how to handle studies with before-after assessments (especially crossover trials), and missing data. More research on this important area is clearly indicated.

Because authors sometimes use a variety of ways of assessing a quantitative outcome, some overviews contain a mixture of studies with binary and continuous outcomes. For such overviews, there was no support for automatic transformation of continuous

outcomes to binary ones, although eight participants felt that this might be acceptable under certain circumstances. The other six felt that this proposal should be studied further before it could be recommended.

Regarding studies with survival time as the endpoint of interest, following a short presentation of some possible approaches by Mahesh Parmar, it was widely agreed (13/14) that useful information could be extracted from published reports of trials with survival as outcome. However, here in particular, it was felt that access to the individual patient data was highly desirable.

## Approaches for Aggregation

Twelve (/14) participants recommend using a statistical test of heterogeneity, and the same 12 also thought that the Cochrane Collaboration should include a test of heterogeneity in its standard software package. A few participants wrote that while some heterogeneity is to be expected, a test is useful for indicating when the underlying heterogeneity is sufficiently large to be detectable, particularly by a test with such low power. Two others recommended a Type I error level of 0.10 rather than the customary 0.05 due to the known low power of the test. A test of heterogeneity should be in the Cochrane Collaboration software.

The questionnaire asked the participants which model they would recommend using if "we have been able to determine that 'substantial' heterogeneity is **not** present in an overview

of trials." Eleven (/14) recommended the fixed effect model and the other 3 responded that either the fixed or random effects would be fine. Two wrote that while either would be fine, the fixed would be preferred because it is simpler to explain. Generally, without substantial heterogeneity, both models would provide similar results. From these results and the discussions during the workshop, everyone agreed that under these circumstances, a fixed effect model should be recommended. Furthermore, the fixed effect model should be the default of the software system.

Considerable disagreement ensued, however, when the discussion turned to the preferred approach under conditions of statistically demonstrable heterogeneity. Both random and fixed effects models had strong proponents, a split which was reflected in the questionnaire responses. One person recommended using neither model and not aggregating results at all, 5 recommended using only the fixed effect, 1 recommended using only the random effects, and 7 recommended using both. A few of those expressing a preference, however, noted that they would also give consideration to not aggregating at all.

Characterising in a few words the differences between the fixed and random effects proponents is challenging. Some of the fixed effect proponents refer to their favoured approach as "assumption-free" and state that it is not directly influenced by any heterogeneity. To the contrary, the random effects proponents state that the fixed effect approach does contain a quite stringent assumption of no differences between the underlying true treatment effects in the individual trials. They contend that a fixed effect method would produce a confidence interval (CI) for an overall effect measure that is

artificially narrow for extrapolation to future individuals, because it does not reflect between-trial variance. A random effects approach takes into account this between-trial variance and produces what they feel is an appropriately larger CI, although the fixed-effect proponents argue that it is too large. Is any common ground discernible in these widely contrasting views?

Some common ground did emerge on at least four points. First and foremost, with substantial heterogeneity, overview analysts should attempt to explore the reasons for the heterogeneity and explain it. While this approach can be most elucidating, the interpretations must be cautious because the analyses are usually post hoc. Subgroup analyses in general were considered to be often useful but problematic in their interpretation.

One potential explanation for heterogeneity, however, can be investigated *a priori*. Differing methodological quality in the component trials could produce bias and, hence, accompanying heterogeneity in an overview. A quality by treatment interaction term may explain away most of heterogeneity. Alternatively, the inclusion criteria for the trials in the systematic overview might be altered to simply exclude the lower quality trials. Obviously, difficulties arise, however, when all of the trials in an overview are of lower methodological quality. Further, assessment of quality is itself controversial. More research on this topic should be encouraged. Whichever approach is used to address heterogeneity, overview analysts should alert readers of their review to its existence and should encourage **cautious** interpretation of the aggregated results.

Secondly, we think everyone agreed that using the fixed effect approach for a test of significance for the overall null hypothesis of no effect in all trials is statistically valid. A statistically significant result would indicate that a treatment effect existed in at least one of the trials.

Thirdly, regardless of whether heterogeneity is present or not, almost everyone agreed that the overall typical effect measure from a fixed effect approach is an informative average measure of treatment effect. (In fact, it will often be very similar to the estimated effect from a random effects approach.) However, the presence of important levels of heterogeneity would alter the interpretation of that average effect.

Fourthly, consensus appeared to emerge that the random effects methods have rather amorphous assumptions that the trials are a random sample from a hypothetical population of trials and that the heterogeneity between trials can be represented by a single variance. Moreover, a pragmatic concern is that the random effects methods give relatively more weight to the smaller studies than the fixed effect methods and smaller trials are often of poorer quality and more subject to publication bias. A consensus emerged that empirical research to assess the importance of these assumptions and the robustness of the random effects approach is necessary.

That leaves as the residual point of dispute the choice of approach to the calculation of a confidence interval around a typical effect measure. Eight of the participants would recommend using a random effects model solely or in conjunction with the fixed effect model. Even more importantly for the Cochrane Collaboration, an even greater majority

(11/14) recommended that calculations from a random effects model at least be available from the software package: 3 recommended fixed effect only, 0 recommended random effects only, 2 recommended both should always be provided, and 9 that fixed and/or random effects should be available as options. Thus, apparently when substantial heterogeneity is present, even given the concerns about the assumptions behind random effects models, a majority of the participants would favour at least examining the approximate allowance that a random effects model affords as preferable to making no allowance at all. A random effects approach should be available in the software.

The entire discussion regarding appropriate approaches for aggregation under conditions of heterogeneity pertained to binary data. The same general principles, however, apply to continuous data. In fact, Peter Armitage recalled that the same discussions about fixed versus random effects models occurred many years ago relative to continuous data in agricultural experimentation.

We should also acknowledge that various fixed and random effects models are available. Generally, not much difference exists between the results from the various fixed effect approaches (Thompson and Pocock 1991). Less information exists on the various random effects approaches. They should be compared in future investigations, particularly the predominant random effects approach (DerSimonian and Laird 1986) with maximum likelihood methods (Raghunathan and Yoichi II 1993).

## Presentation

Although the meeting was not intended to address issues of presentation, these came up in discussion several times and are summarised here (they were not addressed in the questionnaire).

While the odds ratio was the generally preferred effect measure for presenting overviews, several participants suggested that it was not the most helpful way of trying to communicate the overall findings of an overview across to clinicians. Alternatives suggested included the number needed to treat (Laupacis et al, 1988) and simple addition across all trials of the actual numbers of events in the treatment and control groups. More research into the most useful methods of presentation would be welcome.

Some participants felt that presentation of the results of each trial would be enhanced by the inclusion of the weight contributed by each trial to the analysis. (Clearly, when both fixed and random effects models had been used, the comparison of these weights would be of some interest.) There was also some support for the idea of explicit information about the methodological quality of each trial, especially with respect to the nature of the randomisation process.

Other topics mentioned briefly included whether the odds ratios should be plotted on natural or log scales and what level should be used for confidence intervals, both for individual trials and for the overall results. Deeper discussion of issues of presentation at a further meeting is indicated.

## Implications for Consideration

## by the Cochrane Collaboration

(Not necessarily formed through unanimous agreement)

- For binary outcomes, the initial analysis should use the odds ratio.

- In addition to the default odds ratio, the software should provide optional calculations using the relative risk and risk difference.

- The analysis should begin by using a fixed effect model to yield an estimate of the average overall treatment effect and a corresponding confidence interval.

- A test of heterogeneity should be done. If statistically significant (with sentiment in favour of $p<0.1$) heterogeneity is found, the reviewer should consider the possible sources (e.g. differences in dose, timing of treatment, length of treatment, patient characteristics). In particular, the reviewer should explore whether differences in the control of bias among the trials explains the heterogeneity (e.g. method of randomisation, blinding).

- The reviewer should be offered the option, by the software, of performing additional analyses of subsets of trials.

- In addition to the default fixed effect model, the software should provide an option

to calculate effect measures and confidence intervals based upon a random effects model.

- If the heterogeneity cannot be explained, the reviewer should consider the options:
    - Do not aggregate the trials at all
    - Use the fixed effect model with appropriately cautious interpretation
    - Use the random effects model with appropriately cautious interpretation
    - Use both the fixed and random effects models as an additional aid in expressing the uncertainty around the analysis

- For continuous outcomes the approach suggested by Bracken, the weighted combination of differences between means, should be used, unless a preferable approach is forthcoming.

- All analyses should display the weights allotted to each trial.

- Whichever of the above options is chosen, the reviewer should explain clearly what has been done and why.

## References


Bracken MB. Statistical methods for analysis of effects of treatment in overviews of randomized trials. In: *Effective Care of the Newborn Infant,* eds JC Sinclair, MB Bracken. Oxford University Press, Oxford, 1992: 13-18.

DerSimonian R, Laird N. Meta-analysis in clinical trials. *Controlled Clin Trials* 1986;**7:**177-88.

Laupacis A, Sackett DL and Roberts RS. An assessment of clinically useful measures of the consequences of treatment. *N Engl J Med* 1988;**318**:1728-33.

Raghunathan TE, Yoichi II. Analysis of binary data from a multicentre clinical trial. *Biometrika* 1993;**80**:127-39.

Thompson SG, Pocock SJ. Can meta-analyses be trusted? *Lancet* 1991;**338:**1127-30.


## List of Workshop Participants

Douglas Altman; Peter Armitage; Colin Baigent; Jesse Berlin; Michael Bracken; Rory Collins; Kay Dickersin; Diana Elbourne; Richard Gray; Klim McPherson; Andy Oxman; Mahesh Parmar; Richard Peto; Stuart Pocock; Kenneth Schulz; Simon Thompson
